# CUTANEOUS PANCREATIC METASTASIS: A CASE REPORT AND REVIEW OF LITERATURE

**DOI:** 10.4103/0019-5154.44806

**Published:** 2008

**Authors:** H Z Abdel Hafez

**Affiliations:** *From the Department of Dermatology, Assiut University Hospital, Assiut, Egypt*

**Keywords:** *Pancreas cancer*, *skin metastases*, *nodules*

## Abstract

Pancreatic cancer is one of the most dangerous human cancers and will continue to be a major unsolved health problem as we enter the 21^st^ century. This is the case despite advances in imaging technology and surgical management. Indeed, 80% to 90% of pancreatic cancers are diagnosed either at the locally advanced or metastatic stage. Cutaneous metastases originating from pancreatic cancer are relatively rare. The most common site of cutaneous metastasis is the umbilicus, and this is known as the Sister Joseph's nodule. Very few patients have been reported with cutaneous lesions disclosing a pancreatic carcinoma at sites other than the umbilical area. To the best of our knowledge, there have been no previous reports on cutaneous pancreatic metastasis in Egypt. This is a report on a patient with cutaneous pancreatic metastases at the neck and review of reported non-umbilical cutaneous metastases from pancreatic carcinoma in the literatures.

## Introduction

Pancreatic cancer is something to really worry about as it metastasize rapidly.^1^,^2^ Cuatneous metastasis mostly occur around umbilicus.A site other than umbilicus is rarely reported.^3^,^4^

## Case Report

A 55-year-old female who was referred from the oncology department complained of multiple asymptomatic reddish skin nodules at the left side of the neck of 3-week duration.

**Fig. 1:**
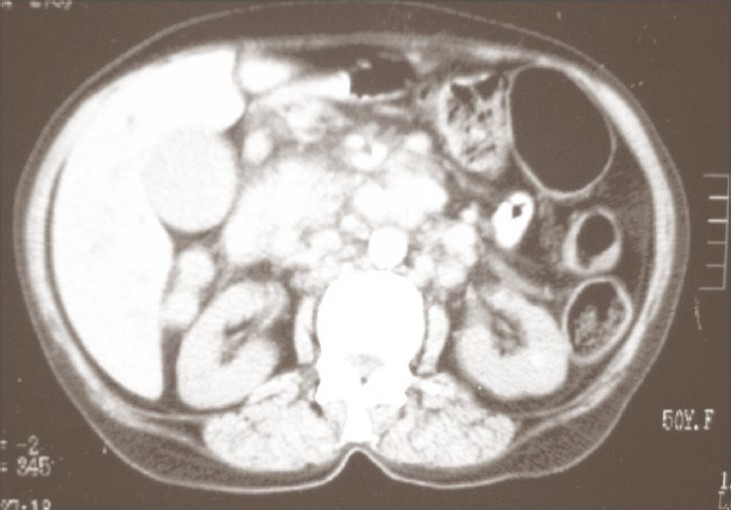
CT scan showing enlarged head of pancreas with heterogeneous soft tissue mass measuring 5 × 5 cm. with multiple porta hepatis and paraaortic lymph nodes with no evidence of hepatic focal lesions

This condition started 6 months before when the patient was admitted because of jaundice and general fatigue accompanied by multiple enlarged firm, nontender left cervical lymph nodes. Laboratory tests showed raised both total and direct bilirubin, raised liver enzymes; hepatitis markers were negative and renal function tests were normal. Chest X-ray was free; abdominal ultrasound showed a mass located at the head of pancreas measuring approximately 4.6 × 4.8 cm (AP × W) with multiple enlarged porta hepatis lymph nodes with evidence of dilated intrahepatic biliary radicals and dilated common bile duct. A computerized tomography scan (CT) of the abdomen revealed the enlarged head of pancreas with heterogeneous soft tissue mass measuring 5 × 5 cm with multiple porta hepatis and para-aortic lymph node enlargements and no evidence of hepatic focal lesions (Fig. 1). Metastases elsewhere were not detected by examination and thorough investigations. Abdominal ultrasound (US) and CT findings were compatible with a cancer of the head of pancreas with multiple metastatic abdominal lymph nodes causing common bile duct obstruction. On abdominal exploration, cholecystojejunostomy and enteroenterostomy were performed; however, the surgeons refused to take a biopsy from the unresectable mass because of the fear of complications arising from a pancreatic fistula.

**Fig. 2:**
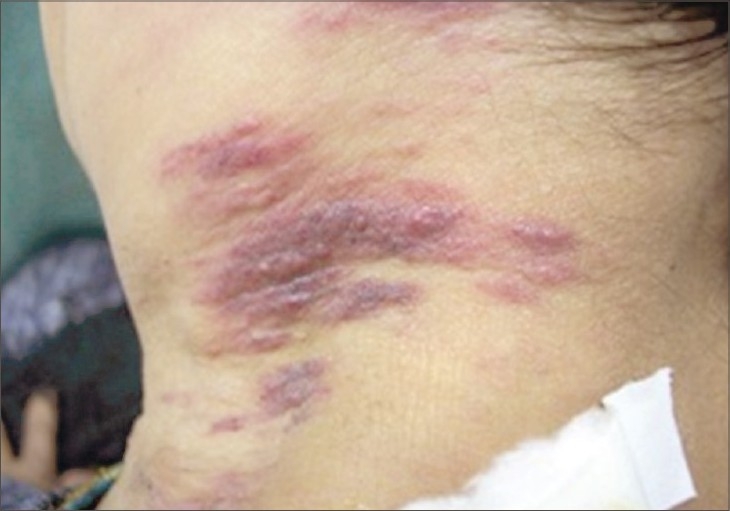
The initial clinical eruption at the left side of the neck

**Fig. 3:**
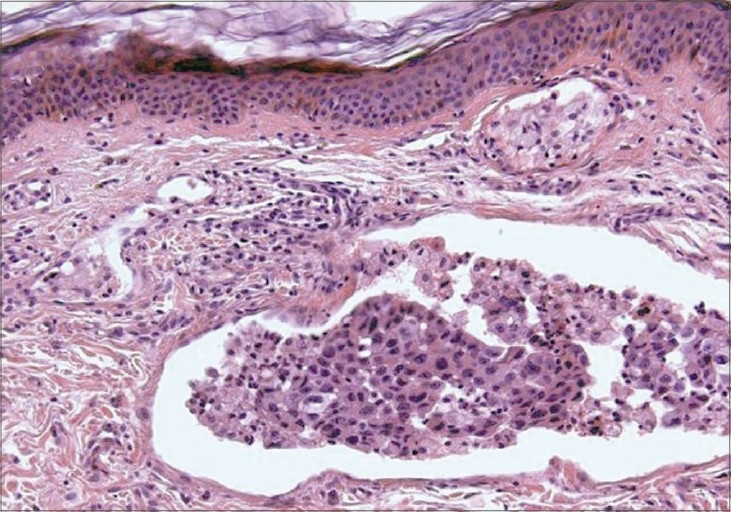
Dermis occupied by numerous tumor nests (H&E ×100 stain)

**Fig. 4:**
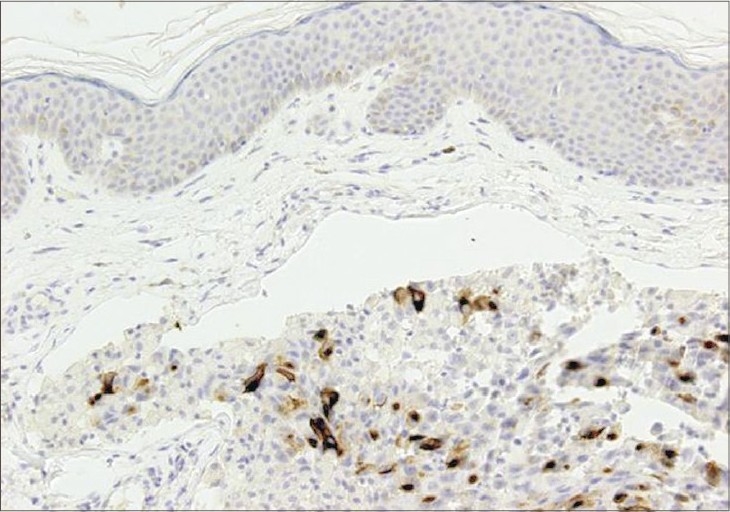
Tumor cells show strong membrane staining (CA 19-9 stain H&E ×100)

Our patient started palliative cytotoxic treatment. During treatment, she developed asymptomatic violaceous nodules and indurated plaques over the skin on the left side of the neck and she was referred to the dermatology department for consultation (Fig. 2). There were no other similar lesions elsewhere on the body. A lymph node biopsy revealed metastatic carcinoma and skin biopsy revealed nests of poorly differentiated atypical cells throughout the dermis (Fig. 3). Silver stain and chromogranin were negative, while EMA was reactive for tumor cells and CA 19-9 was focally positive (Fig. 4). In light of the patient's history of a cancer head of pancreas and the positive immunohistochemical stain result with CA 19-9 for skin biopsy, the diagnosis of a metastatic pancreatic carcinoma was established.

**Fig. 5:**
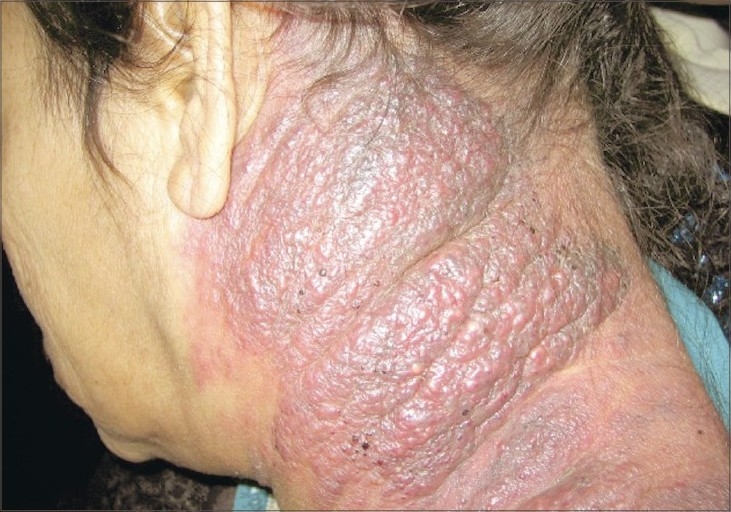
One month later, after receiving the treatment, the reddish, nontender indurated plaques increased in size to cover the entire left side of the neck

One month later, while receiving the palliative cytotoxic treatment, the reddish, nontender indurated plaques increased in size covering the left side of the neck (Fig. 5). At that time, a follow up CT demonstrated a decrease in the size of the pancreatic mass that reached a craniocaudal diameter of 4 cm.

## Discussion

Pancreatic cancer is the fourth leading cause of cancer death. Currently, there are no early diagnostic tests and effective treatment options for this deadly disease.^1^ Morbidity and mortality from pancreatic cancer is conspicuously associated with metastasis; the most frequent sites of metastasis are as follows: lymph nodes, lungs, liver, adrenal glands, kidneys and bones.^2^ Cutaneous metastasis are rare^3^–^4^ and they are generally situated in the periumbilical area.^5^ The mechanism of cutaneous metastasis is not well described; previous studies mostly focused on the "soil and seed" hypothesis. Tumor seeding during resection is a feared complication as recurrence within the peritoneal cavity commonly occurs after resection for curative intent.^6^ Moreover, pancreatic carcinoma is known to metastasize rapidly to the lymphatic system by permeation, embolization, and retrograde spread due to lymphatic obstruction in the pancreas.^7^ Recently, more attention has been focused on the chemotaxis hypothesis, where cancer cells with high expression of chemokine receptor will spread to the specific sites where the legend is highly secreted.^6^ Lookingbill *et al*^8^ reported that cutaneous involvement could occur via three different mechanisms: direct invasion, local metastatic disease or distant metastasis. According to their series, the last mechanism is the least common one, and when it happens, cutaneous lesions arise as multiple nodules grouped in a body area. Takeuchi *et al*^9^ stated that the most frequent cutaneous metastatic site was the umbilicus, distant spread shows that a pancreatic carcinoma can reach all cutaneous tissue via blood or lymphatic systems.

Miyahara *et al*^5^ reported 5 cases and reviewed 17 cases of cutaneous metastasis originating from the pancreatic cancer. In 20 cases, the cutaneous metastases were present prior to the diagnosis of pancreatic cancer. In 11 of these cases, the metastatic lesions in the skin were the first symptoms of pancreatic cancer, and in the other 9 cases, the lesions were discovered by physical examination. They stated that the most common site of cutaneous metastases originating from pancreatic cancer was the umbilicus. Although such cases are rare, it is important to note that metastatic lesions in the skin may be the first sign and one type of distant metastases originating from pancreatic cancer. Horino *et al*^7^ reviewed 49 reported cases of pancreatic metastasis from 1950 to 1999. In the majority of cases, skin metastatic lesions were the first signs of the pancreatic cancer. Moreover, 90.3% of the cases had multiple organ metastases or peritoneal seeding. Only four cases with skin metastases from pancreatic carcinoma are still alive according to the reports. Two of the four cases underwent resection of the pancreas. Their skin metastatic lesions were first noted on physical examination after resection (details were not described). The other two cases underwent chemotherapy (details were not described). After conducting a detailed PubMed search, Yendluri *et al*^10^ reviewed the published English and Japanese literature from the last 90 years. They identified 57 cases of Sister Joseph's nodule originating from the pancreas. Although 70% to 80% of pancreatic adenocarcinomas occur in the head of the pancreas, in patients presenting with a Sister Joseph's nodule, the majority (91%) were in the tail and body of the pancreas. This may relate to the propensity for tail of pancreas cancers to remain asymptomatic until a later stage when distant metastasis has already occurred.

The author, after reviewing the published data, has found 16 cases, excluding our case, with nonumbilical cutaneous metastasis (Table 1). Patients with metastasis to the skin incision or at sites of drain were excluded in this search. Of the 17 cases reviewed (15 men and 2 women), 52.8% of the location sites of primary pancreatic carcinoma were found to be at the head, 23.7% were located at the body and/or tail and for 23.5%, no details were given regarding the site of the primary pancreatic carcinoma. The majority of skin metastasis reported in the literature occurred after palliative procedures, in which the tumor burden remains. In our case, the first skin metastasis was not in the umbilicus, but in the left side of the neck; the metastatic process was confirmed by CT examination, and the primary tumor was found at the head of the pancreas. The focal positive staining of skin biopsy with CA 19-9 supported our diagnosis. Based on the relative frequency of this phenomenon, this case represents a scenario that validates that nonumbilical cutaneous pancreatic metastasis arises secondary to a primary pancreatic cancer located at the head of pancreas.

**Table 1:** Review of cases

Author	Age	Sex	Metastatic site	Tumor site
Sakai *et al*.^11^	47	M	Herpes zoster-like	Head
Taniguchi *et al*.^12^	69	M	Face, head	Head
Taniguchi *et al*.^12^	67	M	Chest, abdomen	No details
Ohhashi *et al*.^13^	79	M	Neck, chest, abdomen	No details
Ohhashi *et al*.^13^	65	M	Back	No details
Sironi *et al*.^14^	72	M	Right thigh	Head
Fukui *et al*.^15^	49	M	Face, chest	No details
Nakano *et al*.^16^	80	M	Occipital scalp	Tail
Miyahara *et al*.^5^	60	M	Face, neck	Body, tail
Miyahara *et al*.^5^	43	M	Scalp	Uncus
Miyahara *et al*.^5^	65	M	Mentum	Uncus
Horino *et al*.^7^	65	F	Chest wall	Head
Ambro *et al*.^17^	63	M	Scalp	Ductal
Florez *et al*.^18^	84	M	Buttock	Head
Takeuchi *et al*.^9^	77	M	Left axilla	Tail
Jun *et al*.^19^	68	M	Right forearm, chest	Body, tail
Our case	55	F	Neck	Head

## Conclusion

Carcinomas of the pancreas represent less than 5% of human malignant neoplasms; skin involvement is rare, and metastasis generally occurs at the umbilical area. We describe an interesting case of cutaneous pancreatic metastasis. To the best of our knowledge, very few patients have been reported with cutaneous metastasis at the neck disclosing a pancreatic carcinoma, making this case particularly interesting. This is the first case of cutaneous pancreatic metastasis reported in Egypt.
